# Integrating Cardiopulmonary Exercise Testing in the Assessment of Aortic Stenosis: A Comprehensive Review

**DOI:** 10.3390/healthcare14030329

**Published:** 2026-01-28

**Authors:** Peter Luke, Helen Banks, Christopher Eggett

**Affiliations:** 1 School of Medicine, Newcastle University, Newcastle upon Tyne NE1 7RU, UK; 2 Freeman Hospital, Newcastle upon Tyne Hospitals NHS Trust, Newcastle upon Tyne NE1 7RU, UK

**Keywords:** aortic stenosis, cardiopulmonary exercise testing, transthoracic echocardiography, stress echocardiography

## Abstract

Aortic stenosis is a progressive and insidious form of valve disease with a high mortality if left untreated. While cardiopulmonary exercise testing is recognised as a safe and effective strategy to aid in the management and timing of valvular intervention, it is often underused due to patient frailty and concomitant comorbidities. This study discusses the pathophysiology of aortic stenosis and current evidence supporting the use of cardiopulmonary exercise testing for the risk stratification and management of aortic stenosis.

## 1. Introduction

Aortic stenosis (AS) is an insidious form of valvular heart disease which is characterised by aortic leaflet fibrosis and calcification resulting in a reduced orifice area and proportional increases in transvalvular gradients [1]. The incidence of AS is strongly correlated with advancing age, affecting around 5% of the population over the age of 65 years, increasing to 9.8% over the age of 80, and is likely to increase 2.4-fold by 2040 and triple by 2060 [2]. It is well established that advanced, untreated AS may cause debilitating symptoms such as shortness of breath, chest pain, and syncope which can detrimentally impact quality of life and increase the risk of mortality [3]. While degenerative calcific AS is most prevalent in the Western world, rheumatic involvement and bicuspid aortic valve (AV) anatomy continue to contribute significantly to disease burden [4,5]. Currently, there are no pharmacological therapies that regress or influence the progression of AS, with surgical or percutaneous valve intervention being the only curative options available. Recommendations provided by the American College of Cardiology and American Heart Association (ACC/AHA) advocate that transcatheter or surgical valve prosthetic valve replacement should be primarily driven by symptoms, with shared decision-making being adopted to consider the overall benefits and risks to the patient [6]. While transthoracic echocardiography (TTE) is the gold standard imaging modality for visualising AV mobility and grading severity, the beneficial role of adopting cardiopulmonary exercise testing (CPET) has been shown to be an effective risk stratification tool [7,8]. This review aims to provide a comprehensive overview of the current evidence supporting the use of CPET in the management of AS.

## 2. The Role of Cardiopulmonary Exercise Testing in Clinical Practice

Cardiopulmonary exercise testing (CPET) is a dynamic, non-invasive technique that evaluates the integrated physiological responses of the cardiovascular, respiratory, and skeletal muscle systems during exercise and simultaneous breath by breath gas analysis [9]. It is widely used for functional assessment in respiratory and cardiovascular disease, investigation of unexplained exertional dyspnoea, monitoring treatment response, and peri- and postoperative risk stratification in patients undergoing major elective surgery [10]. In patients with valvular heart disease, CPET can be used to assess the functional impact of regurgitation and/or stenosis and may be used pre- and post-valvular surgery to assess response to treatment by measuring key variables including oxygen uptake ($\dot{V}$O_2_), carbon dioxide production ($\dot{V}$CO_2_), ventilation ($\dot{V}$E), heart rate (HR), and blood pressure (BP). CPET enables the objective assessment of the physiological systems under controlled stress conditions [11], while allowing clinicians to gain an insight into the pathophysiology of exercise limitation [9]. In a clinical setting, CPET is predominantly conducted on a cycle ergometer as it is regarded as a safe, less weight-bearing modality which is well-tolerated by patients. While cycle ergometry yields fewer motion signal artifacts and noise compared to treadmill-based protocols [9,12], it is recognised that anaerobic threshold (AT) values are often lower compared to those derived from treadmill exercise [13]. The work rate (WR) protocol (Watts min^−1^) is calculated using the patient’s height, weight, sex, and age [14] and the results are compared to “normal” reference values. There are several sets of reference values available and these should be selected appropriately for the patient being assessed [12].

During CPET, expired gases are collected via a facemask or mouthpiece and analysed using breath by breath gas analysis and a differential pneumotachograph [10,13], with continuous monitoring via 12-lead electrocardiography, non-invasive-BP, and oxygen saturation (SpO_2_). In some cases, an arterial line may be used for continuous measurement of arterial blood gases, or an arterial blood gas or capillary blood gas sample may be taken pre- and post-exercise [12,13]. Patients follow a standardized incremental ramp or constant work protocol over a period of approximately 8–12 min [9,12]. Data are collected at rest, during unloaded cycling, throughout incremental loaded exercise until peak, or maximal exercise is reached and during the recovery phase. The data collected throughout the different stages of the CPET provide a comprehensive physiological profile that informs diagnosis, prognosis, and subsequent management. The various protocols and performance of CPET is beyond the scope of this review, but have been well-documented elsewhere [12,15].

**Figure 1:**
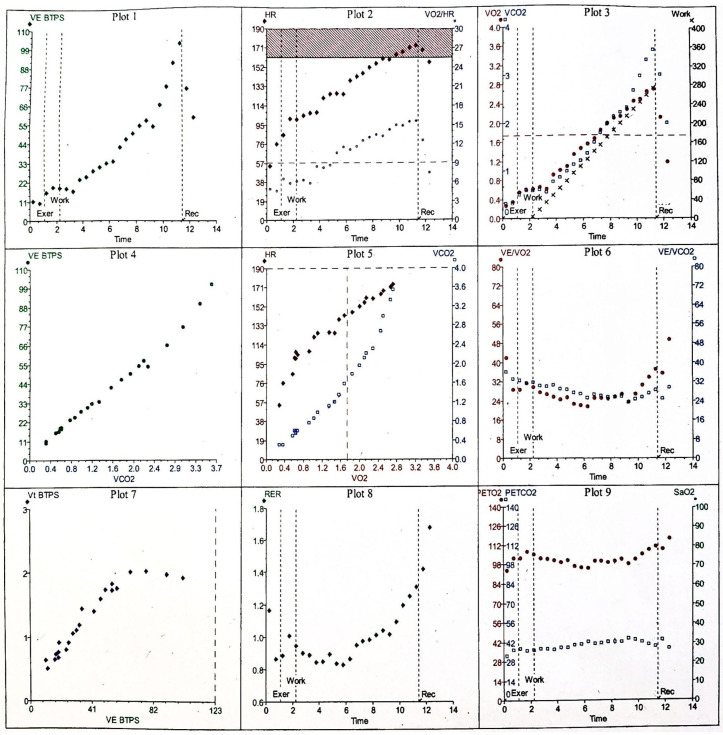
Normal nine-panel plot CPET results of a 30-yr-old female weighing 55 kg. $\dot{V}$E BTPS, minute ventilation at body temperature, ambient pressure saturated with water vapour (L min^−1^); time (min); HR, heart rate (beats min^−1^); $\dot{V}$O_2_/HR, oxygen consumption divided by HR ‘oxygen pulse’ (mL beat^−1^); VO_2_, oxygen consumption (L min^−1^); $\dot{V}$CO_2_, carbon dioxide production (L min^−1^); work (W); $\dot{V}$E/$\dot{V}$O_2_, minute ventilation divided by oxygen consumption (no units); $\dot{V}$E/$\dot{V}$CO_2_, minute ventilation divided by carbon dioxide production (no units); Vt BTPS, tidal volume at body temperature, ambient pressure saturated with water vapour (L min^−1^); RER, respiratory exchange ratio $\dot{V}$CO_2_/$\dot{V}$O_2_ (no units); PETO_2_, end-tidal oxygen tension (mm Hg); PETCO_2_, end-tidal carbon dioxide tension (mm Hg); SaO_2_, refers to arterial oxygen saturation, however in this CPET the peripheral oxygen saturation (SpO_2_) was measured by pulse oximetry and used as a surrogate(%); Exer, unloaded exercise; Work, loaded exercise; Rec, recovery; The dark red box in panel 2 represents the 80–100% range of maximal predicted HR. The horizontal dashed line in panels 3 and the vertical dashed line in panel 5 represent predicted 80% of maximal predicted $\dot{V}$O_2peak_. The vertical dashed line in panel 7 represents the maximum voluntary ventilation.

As CPET provides a comprehensive and often complex profile of physiological response, several systematic and structured approaches to facilitate interpretation of the array of data have been proposed, including the use of a nine-panel plot of the key variables as demonstrated in Figure 1 [10,16]. Optimal interpretation requires the integration of the measured parameters to derive clinically meaningful information to aid clinical decision-making, rather than reviewing parameters independently [13]. The various parameters that are obtained throughout a CPET are discussed below.

Maximal oxygen uptake ($\dot{V}$O_2_ max), expressed both as an absolute value with reference to body weight (kg) and height and as a percentage of predicted reference values, reflects aerobic capacity and is the gold standard measure for cardiovascular fitness and predictor of disease prognosis, morbidity, and mortality in a plethora of diseases [17]. $\dot{V}$O_2peak_ represents the highest oxygen uptake achieved during CPET, whereas $\dot{V}$O_2max_ denotes the point at which oxygen consumption plateaus despite further increases in workload [15]. Attaining $\dot{V}$O_2max_ is typically feasible in physically fit individuals; however, in elderly, deconditioned patients, or those with significant comorbidities, $\dot{V}$O_2max_ is rarely reached, making peak $\dot{V}$O_2_ the more practical metric [10]. Although peak $\dot{V}$O_2_ is widely accepted and routinely employed, existing reference equations are derived from relatively small and homogeneous cohorts [18,19]. Consequently, distinguishing normal from abnormal values can be unreliable, complicating interpretation [17]. To address these limitations, a novel physiological quotient-$\dot{V}$O_2_Q has been proposed as an alternative approach that is independent of reference equations, though further validation is required [17]. Additional criteria used to approximate $\dot{V}$O_2max_ and confirm that a test was truly maximal include: the presence of a $\dot{V}$O_2_ plateau despite increasing workload, a respiratory exchange ratio (RER) exceeding 1.15 (demonstrated in panel 8 of Figure 1, maximum RER achieved during exercise was 1.31), peak exercise blood lactate concentration greater than 8 mmol·L^−1^, attainment of a peak HR ≥ 90% of the age-predicted maximum (shown in panel 2 of Figure 1 by the dark red box). For this 30 yrs old patient, maximum predicted HR was 190 beats min^−1^ and she reached 174 beats min^−1^ (92%), achievement of the predicted maximal work rate (seen in panel 3 of Figure 1 the maximum predicted WR for 30 yr old female was 125 W) and she achieved 275 W (220%), and a Borg rating of perceived exertion (RPE) score of ≥5–6 on the 0–10 scale, corresponding to “hard” to “very hard” effort [11,15]. In patients with severe AS and heart failure, it has been proposed that there is prognostic value in the use of submaximal exercise gas exchange variables such as the oxygen uptake efficiency slope (OUES) as a surrogate estimate for $\dot{V}$O_2max_ [15,20,21,22]. OUES is a derived index based upon the relationship between $\dot{V}$O_2_ and logarithmic converted $\dot{V}$E illustrated as a slope, measured during incremental exertion [23].

**Figure 2:**
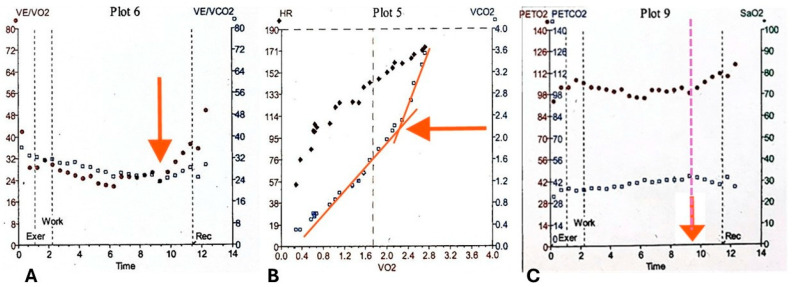
Panels 5 and 6 for determining anaerobic threshold (AT) using the ventilatory equivalents and V-slope methods. For abbreviations, see legend to Figure 1. (**A**) In panel 6, the red filled circles denote the ventilatory equivalents for $\dot{V}$CO_2_ ($\dot{V}$E/$\dot{V}$CO_2_) and the black open squares denote the ventilatory equivalents for $\dot{V}$O_2_ ($\dot{V}$E/$\dot{V}$O_2_). The red arrow indicates where the two slopes crossover and diverge and the anaerobic threshold (AT) can be determined using the ventilatory equivalents. The black dashed vertical lines correspond to Exer, unloaded exercise; Work, loaded exercise; and Rec, recovery; (**B**) In panel 5 the black open squares denote the $\dot{V}$CO_2_/$\dot{V}$O_2_ slope. The 2 purple dashed lines have been manually added to reflect the slope of the curve and identify the point of inflection where there is a sudden increase in gradient demonstrating a shift from aerobic to anaerobic metabolism known as the AT. This method for determining AT is referred to as the V-slope method. The black closed diamonds denote the HR which increases throughout the test. (**C**) Panel 9 can be used to verify the AT identified from panel 5 by using the end-tidal pressure method. The red filled circles show the end tidal O_2_ tension and the black open squares show the end tidal CO_2_ tension. The point of inflection upwards for O_2_ and downwards for CO_2_ has been marked with a verticle purple line and a red arrow and indicates AT The black dashed vertical lines correspond to Exer, unloaded exercise; Work, loaded exercise; and Rec, recovery.

The respiratory exchange ratio (RER), defined as the ratio of carbon dioxide output to oxygen uptake ($\dot{V}$CO_2_/$\dot{V}$O_2_), reflects the relative contribution of carbohydrates, fats, and proteins to energy metabolism [24,25]. During exercise, RER typically rises due to increased glycogen utilization, buffering of lactic acid accumulation, and hyperventilation [11,13]. An RER exceeding 1.15 at peak effort is generally considered indicative of a maximal cardiovascular effort (shown in panel 8, Figure 1), whereas values below this threshold may suggest a submaximal test, potentially limiting the interpretability of the results [10,12,15]. Minute ventilation ($\dot{V}$E) is a measure of the total volume of air entering the lungs per minute calculated by multiplying breathing frequency by tidal volume (V_t_), and is plotted against $\dot{V}$O_2_, $\dot{V}$CO_2_, and WR to provide information on ventilatory efficiency (plots 1 and 4, Figure 1) [26]. At the start of exercise, $\dot{V}$E increases due to an increase in V_t_ which subsequently plateaus in the later stages of exercise, at which point $\dot{V}$E increases due to breathing frequency, as demonstrated in Figure 1 plot 7 by the steady increase in V_t_/$\dot{V}$E until a plateau is reached [15]. Ventilatory equivalents refers to the ratio of $\dot{V}$E to $\dot{V}$CO_2_ ($\dot{V}$E/$\dot{V}$CO_2_) and $\dot{V}$O_2_ ($\dot{V}$E/$\dot{V}$O_2_) and provide an index of ventilatory efficiency, with a lower value representative of more efficient ventilation and exchange of gases [9,26]. The $\dot{V}$E/$\dot{V}$CO_2_ slope displays the interdependent relationship between ventilation and CO_2_ production [26]. AT can be identified using the ventilatory equivalents by observing the point on the curve where $\dot{V}$E/$\dot{V}$O_2_ increases and the slope steepens and diverges from $\dot{V}$E/$\dot{V}$CO_2_, whereas $\dot{V}$E/$\dot{V}$CO_2_ remains on a similar trajectory as during the predominantly aerobic exercise shown in Figure 2. This is due to the disproportionate increase in $\dot{V}$E relative to $\dot{V}$O_2_ [10].

Breathing reserve (BR) represents the residual ventilatory capacity available at peak exercise and is calculated from the difference between the maximum voluntary ventilation (MVV) and the maximum exercise ventilation ($\dot{V}$E_max_). MVV is typically determined prior to commencing exercise either by direct measurement or by estimation once the patient has performed baseline forced spirometry maneuvers estimated using the formula MVV ≈ FEV_1_ × 40 where FEV_1_ denotes the forced expiratory volume in the first second [15]. Under normal physiological conditions, BR should exceed 15–20% of MVV. When $\dot{V}$E_max_ approaches or exceeds 80% of the predicted value and BR falls below approximately 20–25%, this pattern is indicative of ventilatory limitation and may suggest underlying respiratory pathology [15].

Anaerobic Threshold (AT) marks the point during incremental exercise at which the oxygen supply to working muscles becomes insufficient to meet metabolic demand, prompting a transition from predominantly aerobic metabolism to a mixed aerobic–anaerobic state for ATP production. This shift leads to lactate accumulation, which is buffered by circulating bicarbonate, generating additional CO_2_ and causing a disproportionate increase in ventilation and $\dot{V}$CO_2_ as demonstrated in Figure 2 [10]. Respiratory compensation point (RCP) represents a further transition toward predominantly anaerobic metabolism, characterized by an additional rise in ventilation to counteract metabolic acidosis. RCP is typically observed only in highly trained individuals and is rarely emphasized in routine clinical assessment [9]. In CPET, the AT is denoted as a percentage of the predicted value for $\dot{V}$O_2max_ and serves as a key indicator of submaximal functional capacity and for determining exercise prescription [13]. AT can be determined by several methods including the V-slope method, ventilatory equivalents method, end-tidal pressure method and modified V-slope method with the most reliable approach being to integrate several of these approaches [15] as demonstrated in Figure 2

## 3. Normal Cardiopulmonary Response to Exercise

Exercise in healthy individuals elicits a precisely regulated cardiovascular response which involves an increase in cardiac output and peripheral oxygen extraction to meet rising metabolic demands and facilitate carbon dioxide clearance from the working muscles [27]. This response is achieved through a coordinated increase in cardiac output—driven by elevations in HR and SV alongside enhanced venous return, systolic blood pressure, and peripheral vasodilation [28]. Concurrently, respiratory adjustments, including increased Vt and breathing frequency, support efficient gas exchange. The CPET pattern in a healthy individual reflects these physiological adaptations. Figure 1 shows the nine-panel plot results of a normal response to exercise. A maximal test is indicated by one or more criteria: attainment of ≥80% of predicted workload, HR exceeding 90% of the age-predicted maximum, or RER > 1.15 [10,12]. $\dot{V}$O_2_ rises linearly with WR, as demonstrated by the $\dot{V}$O_2_/WR slope as seen in plot 3, Figure 1, which represents the efficiency of converting chemical energy into mechanical work [13]. The AT, determined by validated methods such as the V-slope or ventilatory equivalents approach, should fall within the predicted reference range.

HR increases almost linearly with WR throughout exercise to predicted maximum HR, returning toward baseline during recovery [13]. The VO_2_–work relationship should normally demonstrate a linear response with an increase in $\dot{V}$O_2_ as workload increases at a rate of approximately 10.3 mL O_2_ min^−1^ W^−1^ +/− 1.8 mL O_2_ min^−1^ W^−1^ [10], as shown by measuring the gradient of the curve in Figure 1 plot 3. O_2_ pulse ($\dot{V}$O_2_/HR) rises progressively from the onset of exercise shown in plot 2, Figure 1, reflecting augmented SV and widening arteriovenous oxygen difference, before plateauing near its predicted maximum [10,29]. Systolic BP increases in parallel with $\dot{V}$O_2_, primarily due to sympathetic activation modulating systemic vascular resistance through vasoconstriction and vasodilation to optimize perfusion of working muscles. In contrast, diastolic pressure typically declines slightly as a result of pronounced muscular vasodilation [29]. Ventilation ($\dot{V}$E) increases proportionally with WR until AT, beyond which $\dot{V}$E accelerates sharply, producing a steeper upward curve as shown in plot 1, Figure 1. BR remains >20%, and $\dot{V}$E stays <80% of MVV, indicating the absence of ventilatory limitation [10]. $\dot{V}$E/$\dot{V}$O_2_ and $\dot{V}$E/$\dot{V}$CO_2_ decline during early exercise, reflecting improved ventilatory efficiency, before reversing at AT as shown in plot 6, Figure 1. Beyond this point, $\dot{V}$E/$\dot{V}$CO_2_ rises more steeply than $\dot{V}$E/$\dot{V}$O_2_ due to the disproportionate ventilatory response to CO_2_ generated from lactate buffering [10,15].

## 4. CPET Physiology in Aortic Stenosis

The gradual calcification and subsequent restriction of the AV in degenerative AS leads to left ventricular remodelling as a consequence of increased afterload and wall stress resulting in concentric hypertrophy in accordance with Laplace’s law [30]. LV hypertrophy, as an adaption to mitigate myocardial wall tension and elevated intracavity LV pressure, often has a negative impact upon LV cavity volumes and therefore diastolic function and SV [31]. As the AS progressively worsens, LV myocardial damage ensues with the accumulation of interstitial fibrosis which eventually depresses LV function [32]. Assessment of functional capacity measured by CPET is an invaluable way to identify early cardiac dysfunction in asymptomatic patients, understand the mechanisms behind symptoms, inform prognosis, differentiate cardiac limitation from other comorbidities for preoperative risk stratification, and aid clinical decision-making regarding conservative versus interventional treatment [15,33].

In patients with valvular heart disease, CPET results demonstrate a pattern of cardiac limitation; a combination of LV diastolic impairment along with reduced SV in AS patients with preserved LVEF reduces $\dot{V}$O_2peak_ and O_2_ pulse as a marker of functional capacity [34]. Elevated LV systolic intracavity and diastolic pressures may also have a negative impact upon $\dot{V}$E/CO_2_ visualised in CPET results by an increase in slope of the $\dot{V}$E/$\dot{V}$CO_2_ in plot 5 (Figure 1) and attributed to impairment of alveolar–capillary conductance [35], and worsening pulmonary vascular stiffness [36]. These changes combined with reduced O_2_ pulse as a surrogate of SV shown as a reduced O_2_ pulse curve and early plateauing in plot 2 of the 9 panel CPET result display (Figure 1), leads to a disproportionate rise in ventilation relative to $\dot{V}$O_2_. This is displayed as an elevated $\dot{V}$E/$\dot{V}$O_2_ baseline at the start of CPET and an earlier divergence of the $\dot{V}$E/$\dot{V}$O_2_ (red filled circles) and $\dot{V}$E/$\dot{V}$CO_2_ (black open squares) curves on panel 6 of the 9 panel plot of results (Figure 1, plot 6) and ultimately is responsible for a decline in OUES observed in AS patients [21,37]. Abnormal BP response measured non-invasively throughout exercise can also provide key prognostic information to guide intervention [38]. It is thought that SV cannot be augmented in significant AS and with progressive workloads, may manifest as a blunted or a plateau response observed during exertion. A blunted BP response is defined as a persistent reduction in systolic BP ≥ 20 mmHg, a ≥20 mmHg reduction in BP compared to baseline or the inability to increase systolic BP by 25% compared to pre-exercise BP [39]. The 2025 ESC/EACTS guidelines for the management of valvular heart disease recommend a decrease in BP compared to baseline in asymptomatic AS patients as a class IIa indication for surgical valvular intervention [40]. Interestingly, Nilsson and colleagues recently concluded that in a prospective observational study with 45 symptomatic AS patients, 10% of patients exhibited a drop in BP during CPET with no adverse events recorded [41]. No systolic drop in BP was recorded in these same patients following intervention. A recent study by Carlen et al. [38] also highlighted that the behaviour of systolic BP response towards the end of exertion is also relevant as their results demonstrated greater cardiovascular risk in AS patients with a systolic BP drop (HR: 3.10 (95% CI: 1.85–5.19), while those exhibiting a plateau in BP also demonstrated an increased insignificant cardiovascular risk (HR: 1.17 (95% CI: 0.92–1.48). Table 1 provides a brief summary comparing the normal and AS CPET responses.

## 5. The Role of CPET in Risk Stratification and Management of Aortic Stenosis

AS patients continue to pose diagnostic and management challenges, as decisions regarding surgical AV replacement (sAVR) or transcatheter AV intervention (TAVI) are often guided by a range of TTE criteria (Table 2), including the presence of symptoms. A comprehensive set of indications for AV intervention for symptomatic and asymptomatic AS can be found in the 2025 ESC/EACTS Guidelines for the management of valvular heart disease [33]. It is well recognised that symptoms attributed to significant AS are associated with increased risk of morbidity and mortality [42]. However, many patients with AS are often elderly, frail with a range of co-morbidities, and may unconsciously self-limit their daily activities to avoid precipitating symptoms such as chest pain, dyspnoea and/or syncope [43].

The subtle and gradual modification in physical activity and symptom avoidance behaviour frequently masks true symptom burden and can lead to a delay in sAVR or TAVI referral and an increased risk of post-procedure mortality [43,44]. Conversely, we must acknowledge that not all perceived symptoms observed in AS patients may be directly correlated with valvular heart disease. Concomitant co-morbidities such as chronic obstructive pulmonary disease, a reduced cardiovascular fitness and chronotropic incompetence further complicate the reliable use of symptoms to drive management decisions [45,46,47].

**Table 2:** A consensus grading table for aortic stenosis using British and American Society of Echocardiography recommended guidelines [40,48]. The highlighted column representing ‘very severe’ is exclusive to the British Society of Echocardiography. The American Society of Echocardiography classify severe AS ≥ 4.0 m/s and mean gradient ≥40 mmHg (highlighted in bold) which differs between the two guidelines.

Echo Parameters	Sclerosis	Mild	Moderate	Severe	Very Severe
Peak velocity (m/s)	<2.5	2.5–2.9	3.0–3.9	**≥4.0**–4.9	≥5.0
Mean gradient (mmHg)	-	<20	20–39	**≥40**–59	≥60
Valve area (cm^2^)	-	>1.5	1–1.5	<1	≤0.6
Indexed valve area (cm^2^/m^2^)	-	>0.85	0.60–0.85	<0.6	-
Velocity ratio	-	>0.50	0.25–0.50	<0.25	-

**Figure 3:**
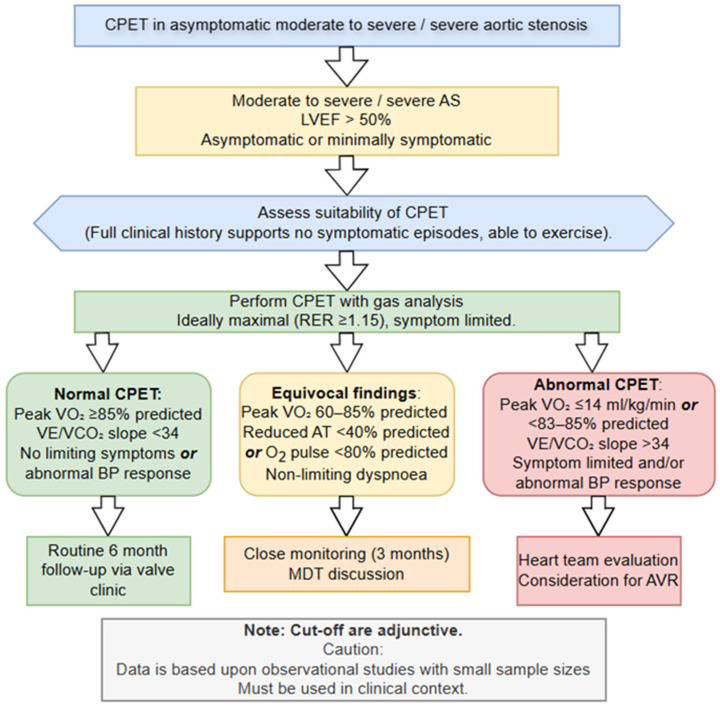
A proposed algorithm which integrates CPET data into the assessment of asymptomatic patients with significant AS. Data supporting this table are derived from studies shown in Table 3.

The management of valvular heart disease guidelines advocate that CPET has a useful prognostic role aiding in the clinical decision-making of asymptomatic AS patients with intermediate severity [33,49]. Table 3 provides a review of primary data and outcomes supporting the use of CPET in asymptomatic severe AS. A proposed algorithm for adopting CPET into AS assessment can be seen in Figure 3. A meta-analysis involving seven studies containing a total of 491 participants concluded that CPET is safe in the severe AS population and is effective at identifying those with an increased risk of adverse cardiac events and premature sudden mortality [50]. A retrospective study conducted by Dhoble and colleagues [34] concluded that while $\dot{V}$O_2peak_ was significantly lower in patients with moderate or severe AS compared to normative values, patients exhibiting a higher $\dot{V}$O_2peak_ (HR 0.83, 95% CI 0.71 to 0.97, *p* = 0.024) and O_2_ pulse (HR 0.80, 95% CI 0.66 to 0.96, *p* = 0.02) observed significantly higher survival rates during a 5 ± 4 year follow-up, regardless of whether participants received valvular intervention. Interestingly, this study also suggested that equivocal symptoms experienced by the AS patients during CPET were not associated with a heightened risk of mortality or intervention compared to those who remained asymptomatic throughout the CPET (*p* = 0.9). Similar findings were observed in a prospective study involving 51 asymptomatic moderate to severe AS with preserved left ventricular ejection fraction of >50% [51]. The results of this investigation demonstrated that the overall $\dot{V}$O_2peak_ was lower than anticipated (21.9 ± 7.4 mL/kg/min) with 57% of patients exhibiting <85% predicted values which were correlated with lower event-free survival compared to normative values (57% ± 11% vs. 93 ± 6%, *p* = 0.036). $\dot{V}$O_2peak_ of ≥85% had a negative predictive value of 97% to develop LV dysfunction or symptoms due to AS. A pilot study involving 43 asymptomatic AS patients confirmed that a VE/CO_2_ slope > 34 (HR 3.68, 95% CI 1.318–10.286; *p* = 0.013) and VO_2peak_ ≤ 14 mL/kg/min (HR 3.06, 95% CI 1.074–8.713; *p* = 0.036) independently predicted an abnormal exercise test and satisfied ESC guideline recommendations for surgical intervention [52].

An investigation by Santos et al. evaluating the feasibility of exercise testing and the potential benefits of sub-maximal CPET in asymptomatic severe AS patients identified that OUES identified patients with reduced functional capacity, with an operator receiver characteristic (ROC) area under the curve of 0.78 (*p* = 0.042) for predicting maximal CPET scores [21]. These findings suggested that OUES may be a useful prognostic marker for those who are unable to perform a maximal exercise test due to frailty. A more recent study by Badiani and colleagues evaluating the CPET parameters such as OUES, O_2_ pulse and $\dot{V}$O_2_ at AT may be a useful in moderate AS patients who are unable to perform a maximal exercise test [22]. This investigation comparing 25 moderate AS with matched controls identified that % predicted OUES (79 ± 15 vs. 89 ± 15%, *p* = 0.013), % predicted O_2_ pulse (89 ± 18 vs. 99 ± 13, *p* = 0.019), and $\dot{V}$O_2_ at AT (14 ± 3.6 mL/kg/min vs. 16.2 ± 3.3 mL/kg/min, *p* = 0.032) were all significantly reduced compared to the control group and emphasises the additive benefit of CPET in risk stratification of AS. Bellander et al. [37] obtained similar findings, suggesting that in a population of 30 symptomatic AS patients that OUES was strongly correlated to peak $\dot{V}$O_2_ prior (r = 0.889, *p* < 0.05) and post- (r = 0.888, *p* < 0.05) surgical valve intervention highlighting that even submaximal CPET is a useful surrogate of functional capacity when maximal CPET is not feasible. Interestingly, when CPET was implemented to assess improvements in cardiovascular fitness post-AS surgical valve replacement, an improvement in median peak workload (133 ± 55 watts vs. 144 ± 67 watts, *p* < 0.001) and ventilatory threshold (1216 ± 391 vs. 1398 ± 309, *p* = 0.001) was observed following intervention. Currently, an ongoing study evaluating the benefits of CPET in 161 AS patients to predict improvement in functional capacity and complications following TAVI is underway which will further promote the role of CPET in this population (ClinicalTrials.gov ID: NCT06833762). Collectively, these findings highlight an adjunctive role for CPET in the assessment and management of AS.

While there is growing evidence supporting the use of CPET for risk stratification in asymptomatic AS, proposed CPET thresholds such as VO_2peak_ and the VE/VCO_2_ slope are largely derived from relatively small, observational cohorts often from single centre or pilot studies. Consequently, questions remain regarding the clinical feasibility and generalisability of CPET within the broader AS population, particularly given the potential impact of selection bias on patient inclusion and subsequent outcomes. Furthermore, the absence of clear, consensus-driven reference ranges can arguably further limit the practical integration and applicability of CPET into routine clinical decision-making.

## 6. The Novel Use of Combined Stress CPET Echocardiography

The role of transthoracic stress echocardiography (SE) in severe AS with reduced LV function has been well established and effectively used to overcome discordant data, differentiate between severe and pseudo-severe AS that can be observed during resting TTE [33,53,54]. There is now growing evidence that SE in those with severe AS with preserved LV function may be beneficial at confirming the true presence of clinically silent AS and provide an early warning for cardiac decompensation [55]. While the use of exercise SE has been embedded into clinical practice and guidelines for some time, there is growing debate about whether combining CPET with SE (CPET-SE) may offer further unique insight into whether a decline in LV systolic function or increases in pulmonary pressures are responsible for any symptoms experienced during exertion [56,57]. This strategy offers the ability to visualise the heart in real-time with the use of suitable acoustic windows while actively performing exercise on a semi-recumbent positional ergometer in conjunction with continuous gas analysis [57,58]. While CPET-SE is relatively exploratory, it offers the ability to evaluate and combine incremental changes observed in TTE parameters such as biventricular function and AV Doppler assessment with CPET metrics such as VO_2peak_, VO_2_/HR and VE/CO_2_ slope to give insight into cardiovascular fitness and subsequent procedural risk [59]. A review by Del Punta and colleagues identified that CPET-SE can be an effective tool to phenotype the pathophysiological mechanism to explain the cause of symptoms for exercise intolerance [56]. In the context of AS, the simultaneous quantification of SV (VO_2_/HR) response during the incremental protocol while further strengthening the case for pulmonary congestion observed on echocardiography by assessing ventilation/perfusion mismatch can be invaluable [56]. CPET-SE also offers the opportunity to capture and analyse sensitive markers of ventricular and atrial function using global longitudinal strain (GLS) at rest and low intensity stages of the CPET protocol. Through speckle tracking of the apical myocardial segments during the low level CPET, a more sensitive evaluation of LV function can be performed beyond the role of LVEF to accurately predict future cardiac events [60,61]. A recent study by Afthonidis et al. evaluating the benefits of CPET-SE in a small group of post-myocardial infarction patients concluded that CPET-SE provided a detailed insight into both functional and myocardial mechanics [62]. The results from this study indicated that a change in GLS was effective at identifying subtle LV dysfunction and these findings correlated with Vo_2peak_ (r = −0.645, *p* = 0.003) and VE/CO_2_ (r = 0.539, *p* = 0.020), further supporting the notion that combining CPET and ESE data provides a more rigorous assessment of cardiac performance [62]. However, we need to interpret the benefits of CPET-SE in the AS population with caution as there are very few peer review publications available and where applicable, relatively small sample sizes reduce the generalisability of these findings. There are also questions around the practicality of CPET-SE, reproducibility and expertise to interpret the overall findings provided by the combined modality. Ultimately, patients most likely to benefit from CPET-SE are those with asymptomatic or equivocal severe AS, low-flow low-gradient AS, and individuals with disproportionate or unexplained symptoms. In these subgroups, CPET–SE may offer a complementary physiological and haemodynamic overview that improves symptom detection, risk stratification, and optimise timing of AV intervention beyond resting assessment alone. As BP is recorded throughout a CPET-SE, there is the ability to combine GLS with systolic BP to derive non-invasive myocardial work (MW). TTE-derived MW is recognised as a comprehensive assessment of cardiac mechanics and energetics which is relatively afterload independent and has been shown to reflect myocardial oxygen consumption when compared to positron emission tomography [63,64,65]. Limitations for adopting GLS in CPET-SE include suboptimal image quality at rest and with increased respiration rate when exercising. A further consideration is higher frame rates CPET-SE due to the inevitable increases in HR [66]. Studies that have performed GLS following CPET-SE reportedly use 70 frames per second (fps) as opposed to the recommended frame rate of 40 fps used in resting TTE [63]. The adoption of GLS and MW are potential areas of future research that could offer more sensitive risk stratification for those with asymptomatic significant AS.

## 7. Conclusions

CPET is an invaluable tool for risk stratification and assessment of functional capacity and symptoms of patients with valvular heart disease including AS. While referrals for CPET in the AS population remain relatively low due to patient comorbidities and frailty, CPET is feasible and safe with emerging evidence supporting that even submaximal CPET can be useful to risk stratify patients prior to intervention. The use of CPET-SE in the AS population may provide further evidence of LVEF degradation and insidious increases in pulmonary pressures which can support CPET parameters. Further studies are required to confirm reference ranges when adopting CPET when risk stratifying AS patients. Additional attention should evaluate the complementary role and potential benefits of incorporating GLS and MW during CPET-SE to ascertain if these TTE-derived measurements provide better prognostic outcomes for AS patients.

## Figures and Tables

**Table 1:** Summary table comparing normal and AS key CPET variables. Abbreviations—see Figure 1 legend.

Parameter	Normal CPET Response	AS CPET Response
$\dot{V}$O_2peak_	>80% maximum predicted	Reduced <80% maximum predicted
$\dot{V}$O_2−_ WR relationship ($\dot{V}$O_2_/WR)	Linear response 10.3 mL O_2_ min^−1^ W^−1^ +/− 1.8 mL O_2_ min^−1^ W^−1^	Reduced <8.7 mL O_2_ min^−1^ W^−1^ +/− 1.8 mL O_2_ min^−1^ W^−1^
AT	>40% $\dot{V}$O_2peak_	Reduced <40% $\dot{V}$O_2peak_
O_2_ pulse	>80% maximum predicted	Reduced <80% maximum predicted
$\dot{V}$E/$\dot{V}$CO_2_	<34 L^−1^ L at AT	Elevated
BP	Increases throughout test	Blunted response/reduces throughout test

**Table 3:** Primary studies evaluating CPET parameters in patients with significant AS.

Study	Population/Methods	Findings
CPET vs. exercise echo in asymptomatic AS [51].	51 asymptomatic moderate to severe AS (V_max_ > 3 m/s, LVEF > 50%) patients underwent CPET vs. supine exercise echo.	A low VO_2peak_ (<85% predicted) was observed in 57% of the cohort with a higher likelihood of lower event survival compared to normal VO_2_ response. A VO_2peak_ ≥ 85% was associated with a negative predictive value of 97%
The value of CPET in asymptomatic AS [52].	A pilot study, 43 severe AS patients without reported symptoms performed CPET.	$\dot{V}$O_2peak_ ≤ 14 mL/kg/min and $\dot{V}$E/$\dot{V}$CO_2_ slope > 34 were independently associated with abnormal exercise responses and reaching guideline surgical class I triggers.
Submaximal parameters in the assessment of AS [21].	25 severe asymptomatic AS patients with submaximal CPET.	OUES identified functional limitation where traditional peak measures were not obtainable highlighting the relevance and benefit of submaximal parameters.
Comparison of CPET data in AS patients pre- vs. post valve replacement [37].	30 severe AS patients prospectively performed maximal CPET with a RER > 1.05 pre- and post-valve replacement.	No significant difference was observed in mean $\dot{V}$O_2peak_ between pre- and post-AV replacement. OUES was significantly correlated with VO_2peak_ in pre- and post-valve replacement groups.

## Data Availability

No new data were created or analyzed in this study.
